# Predicting quality of life with physical and cognitive functioning among older adults with cognitive impairment

**DOI:** 10.1093/geroni/igab046.2611

**Published:** 2021-12-17

**Authors:** Rhayun Song, Moonkyoung Park, Jisu Seo, Fan Xing, Yuelin Li, Ahyun Ryu

**Affiliations:** Chungnam National University, Dae Jeon, Taejon-jikhalsi, Republic of Korea

## Abstract

Purpose: Older adults experience abnormal declines in physical and cognitive functioning that increase their risk of dependence, subsequently quality of life. This study aims to explore the relationship between physical and cognitive functioning, and to predict quality of life among older adults with mild cognitive impairment. Methods: Survey was conducted with older adults registered at dementia support centers. Seventy-four older adults signed the consent form and participated in the study. physical functioning consisted of grip strength, balance (OLS), Timed up and go, and activities of daily living. Cognitive functioning was measured by K-MOCA. SF-12 was used to assess quality of life. Results: The participants was 76 years old on average, more women (75.4%), and mostly elementary or less education level (60.9%). Physical functioning explained 22.1% of variance in cognitive functioning after controlling for age and gender (F change=4.789, p=.002). Balance (OLS: t=2.304, p=.024) and grip strength (t=2.207, p=.031) was significant predictors. Physical and cognitive functioning explained 36.7% of variance in quality of life after controlling for age and gender (F =5.466, p<.001). Indicators of physical functioning, TUG (t=-3.252) and grip strength (t=-2.633), were the most significant predictors of quality of life, while cognitive function explained additional 3.1% of variance in quality of life (F=3.216, p=.078). Conclusion: Physical functioning were significant predictors of cognitive functioning, subsequently to quality of life among older adults with cognitive impairment. Health promoting strategies should focus on improving physical functioning of this population to maintain or prevent cognitive declining, and to promote quality of life.

